# Cardiac tamponade secondary to empyema

**DOI:** 10.1002/ccr3.2684

**Published:** 2020-02-05

**Authors:** Naoki Kawakami, Kohei Kushimoto, Fumitake Saito, Ho Namkoong

**Affiliations:** ^1^ Department of Emergency and Critical Care Medicine St. Luke's International Hospital Tokyo Japan; ^2^ Department of Pulmonary Medicine Eiju General Hospital Tokyo Japan; ^3^ Laboratory of Clinical Immunology and Microbiology National Institute of Allergy and Infectious Diseases NIH Bethesda MD USA

**Keywords:** cardiac tamponade, empyema

## Abstract

A 70‐year‐old male presented with a 1‐month history of malaise and fever. He was diagnosed with chronic empyema and went into shock by cardiac tamponade after treatment. This case shows that even chronic empyema can cause cardiac tamponade. It should be considered as differential diagnoses of shock after empyema treatment.

## Graphical abstract

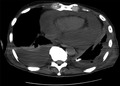

## 1 PICTURES IN CLINICAL MEDICINE

**Figure 1:**
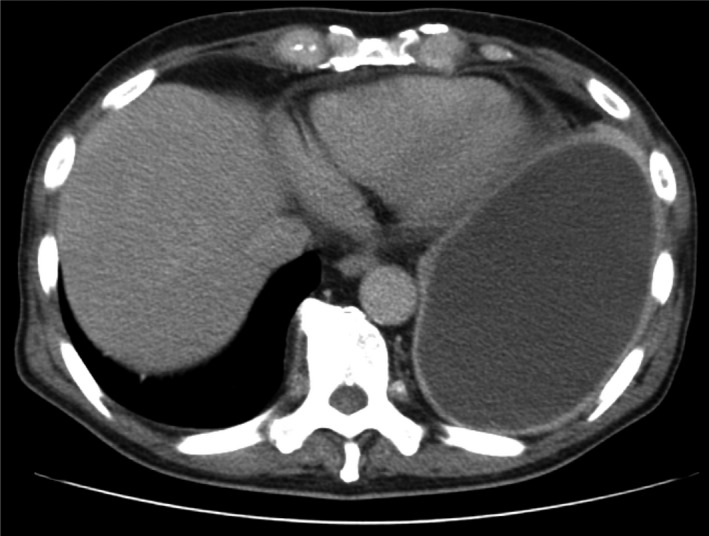
Enhanced chest computed tomography scan obtained at admission, showing extensive encapsulation of the left lung with thickened pleura

**Figure 2:**
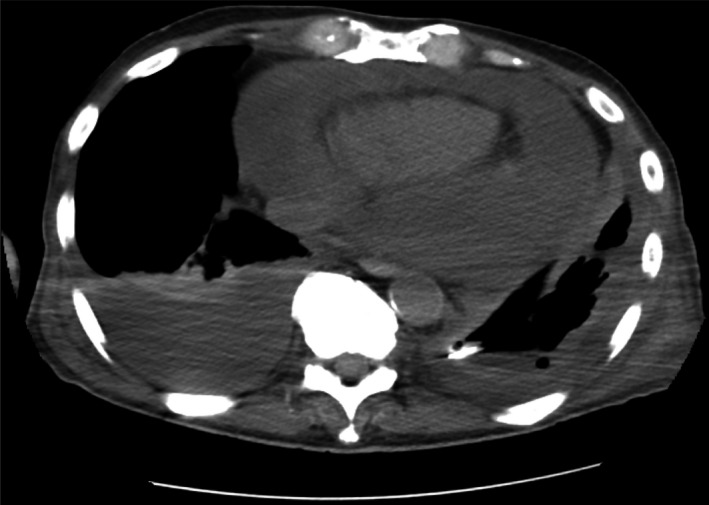
Enhanced chest computed tomography scan obtained at 6 hospital days, showing markedly increased pericardial effusion in the pericardial space, indicating cardiac tamponade

A 70‐year‐old male smoker presented with a one‐month history of malaise and fever. Enhanced chest computed tomography (CT) revealed extensive encapsulation of the left lung with thickened pleura (Figure 1). Pleural fluid culture was positive for *Fusobacterium nucleatum*. Initially, inserting a drainage tube and antimicrobial therapy improved his clinical course. However, 5 days after treatment initiation, he suddenly went into shock with loss of consciousness. Repeated CT revealed markedly increased pericardial effusion in the pericardial space, indicating cardiac tamponade (Figure 2). We inserted a pigtail tube into the cardiac sac for surgical drainage. Pericardial effusion was purulent and had low glucose concentrations, although its culture was negative. Four weeks later, he recovered from empyema and cardiac tamponade.

Extensive chronic empyema possibly caused secondary infection and inflammation of the pericardial sac and progression to cardiac tamponade. Although acute empyema sometimes rapidly evolves into cardiac tamponade,^1^, ^2^ this case is intriguing in terms of showing that even chronic empyema can also cause cardiac tamponade a few days after standard treatment. We should consider cardiac tamponade in the differential diagnoses of shock after empyema treatment especially when it is located on the side of mediastinum.

## AUTHOR CONTRIBUTIONS

All authors participated in the review of the manuscript. NK: drafted the manuscript. KK, FS, and HN: participated in the data collections. FS: supervised the project. HN: drafted the manuscript. All authors read and approved the final manuscript.
